# Sectioning on the street – futility or utility?

**DOI:** 10.1192/pb.bp.115.052449

**Published:** 2016-12

**Authors:** Philip Timms, Jennifer Perry

**Affiliations:** 1 South London and Maudsley NHS Foundation Trust, London, UK

## Abstract

**Aims and method** A service evaluation was undertaken to examine outcomes in patients who were street homeless (‘rough sleepers’) and who were compulsorily admitted to hospital under the Mental Health Act 1983. The data were collected from the patients' case notes.

**Results** At 1-year follow-up, patients had positive outcomes in areas such as accommodation status, registration with a general practitioner and engagement with the clinical team.

**Clinical implications** The study shows that the intervention of a Mental Health Act assessment and compulsory hospital admission in homeless people on the street is associated with positive outcomes at 1 year.

Homelessness is known to be both a cause and a consequence of mental illness,^1,2^ but schizophrenia is the only psychiatric disorder explicitly associated with homelessness (referred to as ‘vagrancy’) in the ICD-10 classification of diseases.^3^ Evidence from different countries gathered over the past 5 decades supports this. Rates of psychosis are 4–15 times higher among the homeless population,^4^ and 50–100 times higher among the street homeless (those who sleep outside: on the street, in parks or in other open spaces),^5^ compared with the general population. Such populations have been shown to also have unmet physical healthcare needs,^6^ higher mortality rates^7^ and higher rates of personality disorder, self-harm and drug and alcohol misuse.^5^

The background level of homelessness is increasing. Rough sleeping in England has grown by over 50% since 2010, with an estimated 2744 people sleeping out on any one night in 2014.^8^ The level of street sleeping in London has more than doubled since 2009/2010, with 7581 sleeping out at some point during the year 2014/2015.^9^

Assertive outreach has been shown to be an effective model for engaging homeless people with mental health services.^10^ In areas with high numbers of homeless people, specialist mental health outreach teams have been established. These teams work closely with voluntary sector accommodation and street outreach teams. In spite of the active efforts of such services, a small proportion of people remain on the street because, actively or passively, they refuse help. Their extreme degree of isolation and self-neglect often suggests the presence of a mental disorder and results in a request for an assessment for compulsory admission to hospital. Such assessments are time-consuming, expensive and potentially distressing for the person involved. To continue such an intervention, we really need to be sure that we are doing more good than harm.

## Method

The aim of this study was to establish whether the intervention of a Mental Health Act assessment leading to hospital admission is effective in helping rough sleepers with mental illness.

We identified our study group from two patient lists belonging to the South Thames Assessment Resource and Training (START) team, one of the surviving specialist services for homeless people in London. The first was a paper list of referrals to the START team, which covered all patients referred between November 2010 and December 2012. The second was a paper list of patients assessed under the Mental Health Act by the team's approved mental health professionals (AMHPs) from 2007 to 2013. To be included in the study, the person had to:

- be an established rough sleeper (minimum of 1 month rough sleeping)
- have had a Mental Health Act assessment leading to hospital admission under a section of the Mental Health Act
- have been discharged from hospital
- have left hospital for 1 year or more, or have been appropriately discharged to their general practitioner (GP) within 1 year.

We then examined the case notes of those meeting the criteria to establish their demographic data, duration of homelessness, diagnosis and several proxy indicators of outcomes 1 year after they were discharged from their index admission to hospital. One of these (fairly crude) measures was of engagement, assessed by reviewing the notes over the 1-year period/up until the point of discharge. ‘Well engaged’ indicated there were no documented problems with attendance at community team appointments, ‘partially engaged’ indicated there were some documented problems, and ‘no engagement’ meant there was no documented engagement of the patient at follow-up.

Medication adherence was also crudely assessed from the notes. ‘Good’ indicated there were no documented problems with adherence, ‘partial’ indicated there were some documented problems and ‘none’ indicated that the patient had completely stopped taking their prescribed medication.

The Combined Homelessness and Information Network (CHAIN) database^9^ (a London-wide database of hostel use and contact with street outreach teams) was consulted to confirm information regarding the length of homelessness history.

Positive outcomes at follow-up were considered if patients were housed, registered with a GP, adherent to their medication, engaged in employment and attending appointments either with the specialist mental health outreach teams or local community mental health teams.

## Results

We identified 32 individuals meeting the study criteria.

### Demographic data and background

**Fig. 1:**
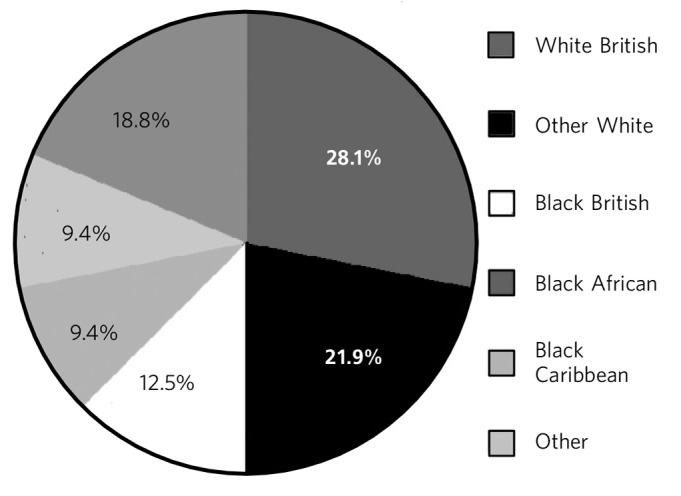
Patient ethnicity

The gender ratio was 4:1 male to female, and a mean age was 44 years (range 24–84). Half (50%) of the group were White, nearly a third (31%) were Black and 41% were British nationals (Fig. 1). Apart from one person with alcoholic dementia, all had a diagnosis of psychosis and 44% had had a previous mental health hospital admission.

**Fig. 2:**
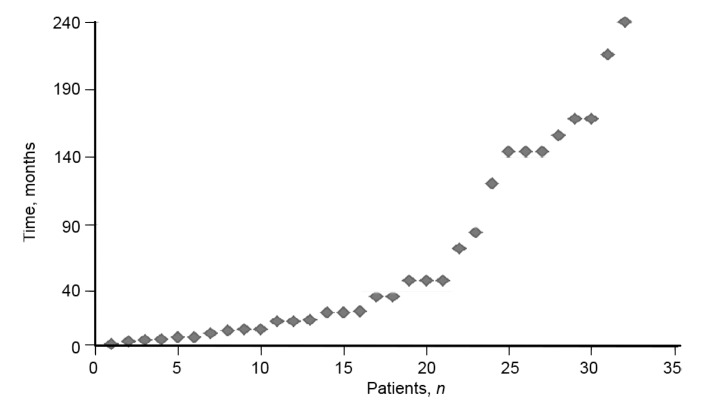
Duration of rough sleeping

The duration of homelessness ranged from 4 weeks to 20 years (Fig. 2). Evidence shows that homeless people are less likely to access services^5^ and thus engage in the treatment they need for their mental health conditions. As mental health problems increase, evidence suggests that individuals may be even less likely to access services.^11^ It is therefore reasonable to assume that, for most of their period of homelessness, the participants had received no consistent or effective treatment, and that many had long durations of untreated psychosis.

### Admission data

All 32 patients were admitted under Section 2 of the Mental Health Act 1983 for assessment and 9 (28%) were then transferred to Section 3 to allow longer-term treatment. The median length of hospital admission was 60 days (range 6–360).

#### Repeat admissions

A third of the study group (*n* = 11, 34%) had a subsequent hospital admission during the following year and 2 patients were in hospital 1 year after discharge. Nearly half of this group (45%) were discharged directly to the street after their first admission, but of the 21 with no subsequent admission, only 2 were discharged to the street. Seven patients with repeat admission (64%) were discharged without medication, in contrast to only 4 out of the group without a further admission (19%). Four patients (36%) in the repeat admission group were not given a diagnosis by the in-patient team. On repeat hospital admission, three of these patients were diagnosed with psychosis, treated and given accommodation. One patient was not treated or given accommodation, but the START team continued to feel that she had a mental illness that warranted treatment.

### Outcomes at 1 year/point of appropriate discharge to GP

#### Engagement with CMHT

Two patients were discharged to their GP at 11 months as they were stable and doing well. Two patients were lost to follow-up: one patient went AWOL from his accommodation and the team lost contact with him at 6 months, and one patient was discharged to their GP at 8 months as they refused to engage. At 1 year follow-up, two patients were back in hospital.

We assessed subsequent engagement with community teams by using the crude measure of attendance at appointments over the 1 year period after discharge: 50% were well engaged, 44% were partially engaged and 6% were not engaged with the team over the 1 year period/at the point of discharge.

The two patients who were lost to follow-up have been included in our analyses and their outcomes at point of loss to follow-up have been used.

#### Accommodation

The majority of patients in the study (*n* = 26, 81%) were living in accommodation at follow-up, mostly supported accommodation (Table 1).

**Table 1:** Types of accommodation at 1-year follow-up

Type	Patients, *n*
Supported accommodation	18
Shared accommodation, no support	1
Residential care home	1
Flat	6^a^
Homeless	6

a. Two flats were funded by the No Recourse to Public Funds Panel, a local panel

#### GP registration

Before admission to hospital, 38% of the group (*n* = 12) were registered with a GP, but this increased to 78% (*n* = 25) at follow-up.

#### Employment

At the point of hospital admission, one patient was employed in voluntary work. At follow-up, four patients were doing voluntary work but none were in paid employment.

#### Medication adherence

Patients' adherence to medication was assessed over the 1-year period: 22% (*n* = 7) had good adherence, 41% (*n* = 13) had partial adherence, 22% (*n* = 7) were non-adherent and 16% (*n* = 5) were not prescribed medication.

## Discussion

This retrospective study suggests that compulsory hospital admissions of patients with mental illness who are rough sleepers can have broadly positive outcomes at 1 year. There seem to be clear gains in accommodation status, GP registration, attendance at appointments and medication adherence. The benefits in terms of employment are marginal (4 patients were in voluntary employment and the remaining 81% (*n* = 26) remained unemployed at follow-up), however, this is in line with literature which shows that the employment rate of patients with mental illness, regardless of their accommodation status, is low.^12^

Eleven patients had repeat hospital admissions; 4 of these patients were initially discharged to the streets without medication and without a diagnosis. The majority of this group (*n* = 3) were re-admitted fairly quickly (again using the Mental Health Act to compel admission) and then subsequently treated. This may reflect our clinical experience that ward teams can sometimes have difficulty in recognising the functional severity of disorders where the patient is not violent, overtly distressed or agitated. This does perhaps suggest an argument that homeless teams should have their own hospital beds, or at least a single ward for such admissions. This would enable a body of shared experience and expertise to be built up in dealing with this group of patients.

Despite this small study showing positive outcomes, some dilemmas and difficulties remain.^13,14^ Ethical dilemmas arise with the uncomfortable notion of forcing a service upon a person who does not want it when they are not in immediate danger. In normal practice, such invasion of an individual's autonomy is justified by the context of a detailed long-term knowledge of their psychiatric history, but such background knowledge is often absent in those seen on the street.

The experience of the authors is that these assessments are uncomfortable. For the patient, the interruption of their established *modus vivendi* can be very distressing. They will often have isolated themselves in response to threatening psychotic symptoms, or may experience an ill-articulated but profound discomfort with other people. It is also difficult for the assessors. The police do have powers to apprehend a person who seems to be unwell in a public place, however these are usually only exercised if the person seems to be an immediate risk to themselves or others. As a result, the person cannot be moved to a safer, more contained space and so the assessment is done *in situ*: in the street, in the park, in the stairwell or doorway. There is little sense of privacy or containment, particularly as the proceedings are sometimes in full view of passers by, who may or may not take an interest in what is happening. It is often physically uncomfortable: one is not sitting down but bending or squatting and, according to the season or time of day, can be cold, windy and wet. Within the UK legal framework, formal Mental Health Act assessments are time consuming and costly. Two independent doctors, an AMHP, the ambulance service and (usually) the police all have to attend simultaneously. Coordinating five different agencies is fraught with difficulties and it is not uncommon for one participant to cancel at the last minute, resulting in the cancellation of the whole assessment.

Statistics show that the majority of people sleeping rough in London are male (86%) and White (69%); 57% are aged between 26 and 45 years, 12% are under 25 and 10% are over 55. Where nationality was recorded, 43% (3212) of those sleeping rough were UK nationals and 36% were from Central and Eastern European countries.^9^ These data show that our sample was representative of the age, gender and ethnicity of those who sleep rough in London.

There were several limitations to this study. The sample size was small and the outcome measures were crude and did not reflect the patient experience of this intervention. It is difficult to say how complete the AMHP's paper list of patients assessed under the Mental Health Act was, as it relied on all of the team's AMHPs regularly updating it. Another limitation was that there was no comparison group with a domiciled population.

This group of entrenched rough sleepers with psychosis, and (by implication) often long duration of untreated psychosis, were generally well or partially engaged with services at 1 year. These findings are consistent with those of a small London study in 1999, which looked at 12 people with psychosis who were sleeping out.^15^ After compulsory admission to a mental health ward from the street, 11 were still accommodated and in touch with mental health services at follow up (median of 21 months).

Even with their limitations, these findings should go some way to counter a therapeutic nihilism that we have sometimes encountered towards both homeless people and people with long duration of untreated psychosis.

In terms of future study, we plan to undertake a further evaluation to look at a larger group of individuals over a 2-year period, using validated outcome measures (such as the routinely collected Health of the Nation Outcome Scales). We hope also to conduct a qualitative study to elicit the patients' view of this intervention.
